# Transient bradycardia during ^177^Lu-DOTATATE therapy: A clinically manageable phenomenon with increased risk in patients with cardiac enlargement

**DOI:** 10.1007/s12149-025-02150-4

**Published:** 2025-12-29

**Authors:** Hirofumi Yamada, Kota Yokoyama, Junichi Tsuchiya, Miki Miura, Eriko Katsuta, Keiichi Akahoshi, Tomomi Akiyama, Keisuke Takino, Daisuke Ban, Ukihide Tateishi

**Affiliations:** 1 Department of Diagnostic Radiology, Institute of Science Tokyo, Bunkyo-ku, Tokyo, Japan; 2 Department of Hepato-Biliary-Pancreatic Surgery, Institute of Science, Tokyo, Bunkyo-ku, Tokyo, Japan; 3 Department of Nursing, Institute of Science Tokyo, Bunkyo-ku, Tokyo, Japan; 4 Institute of Science Tokyo Hospital. Radiology Center, Bunkyo-ku, Tokyo, Japan

**Keywords:** ^177^Lu-DOTATATE, PRRT, Neuroendocrine tumor, Bradycardia

## Abstract

**Objective:**

Lutetium (¹⁷⁷Lu) oxodotreotide (¹⁷⁷Lu-DOTATATE, Lutathera^®^) is widely used for neuroendocrine tumors. Although cardiovascular adverse events are not well recognized, we frequently observe bradycardia during infusion under monitoring. We aimed to characterize heart-rate changes during ¹⁷⁷Lu-DOTATATE administration and explore risk factors for bradycardia.

**Methods:**

We retrospectively reviewed 130 administrations in 37 patients (April 2022–April 2025). Heart rate was recorded at four time points: (i) upon entering the room, (ii) the start of ¹⁷⁷Lu-DOTATATE administration (during L-lysine/L-arginine amino acid infusion), (iii) dose-rate adjustment at 10 min, and (iv) the end of infusion at 30 min. Repeated-measures ANOVA was used to evaluate changes in vital signs. Demographics, comorbidities, and laboratory values (electrolytes, hemoglobin, albumin, total protein) were collected. Cardiomegaly on imaging was determined visually using recent CT or MRI. Multivariable analyses were performed to examine predictors of heart rate change. Patient characteristics were also compared between those with and without bradycardia (≤ 50 bpm) using the chi-squared test.

**Results:**

Mean heart rate decreased over time: upon entering the room (72.3 bpm), at the start of ¹⁷⁷Lu-DOTATATE administration (64.5 bpm), and during dose rate adjustment (59.5 bpm). (all *p* < 0.001). Furthermore, at the end of infusion (66.2 bpm), it returned to a level similar to that at the start of ¹⁷⁷Lu-DOTATATE administration; patterns were similar across treatment cycles. Mean blood pressure decreased upon initiation of ¹⁷⁷Lu-DOTATATE (119.3/73.6 mmHg) compared to upon entering the room (127.8/78.3 mmHg), with no differences observed at other time points. Twenty-two administrations reached ≤ 50 bpm; chest discomfort occurred in five instances and resolved with observation. Multivariable models showed no significant predictors for the magnitude of heart-rate change. However, cardiac enlargement was associated with bradycardia during rate adjustment (*p* = 0.045). No significant associations were seen with age, sex, history of arrhythmia, or laboratory parameters.

**Conclusions:**

Transient heart-rate decline, occasionally meeting bradycardia, commonly occurs during ¹⁷⁷Lu-DOTATATE infusion but typically resolves by completion and rarely requires intervention. Recognizing its benign, self-limiting nature—and the higher susceptibility among patients with preexisting cardiac enlargement—may reduce unnecessary treatment interruptions and improve procedural safety.

**Supplementary Information:**

The online version contains supplementary material available at 10.1007/s12149-025-02150-4.

## Introduction

Peptide receptor radionuclide therapy (PRRT) with lutetium (¹⁷⁷Lu) oxodotreotide (¹⁷⁷Lu-DOTATATE, Lutathera^®^) is widely used for the treatment of neuroendocrine tumors (NETs) in various organs [1–3]. Nausea and vomiting have been identified as common adverse events associated with ¹⁷⁷Lu-DOTATATE, occurring in approximately 59% and 47% of patients, respectively, in the NETTER-1 trial, although most cases were mild to moderate and attributable to concomitant amino acid infusion [1]. In contrast, cardiovascular adverse events have been reported with lower frequency; in the NETTER-1 trial, only flushing was documented (13%) [1], and the FDA’s comprehensive Full Prescribing Information also references alterations in blood pressure of various grades (14%) and atrial fibrillation (5%) in clinical studies [4], however, there is no mention of changes in heart rate itself.

In a similar manner, in the U.S. post marketing surveillance , solely hypersensitivity reactions, encompassing angioedema, were reported, with no mention of alterations in heart rate itself.

However, based on our clinical experience, when ^177^Lu-DOTATATE is administered under electrocardiogram (ECG) monitoring, patients often experience transient decreases in heart rate, sometimes dropping below 50 beats per minute. The objective of this study was to ascertain whether heart rate undergoes a decrease prior to, during, and following ^177^Lu-DOTATATE administration, and under what circumstances heart rate is more likely to decrease. Furthermore, we aimed to evaluate the clinical implications of these findings, including points that require attention in practice and the extent to which interventions are necessary, in order to provide guidance for managing similar situations.

## Materials and methods

### Study design and patients

A retrospective review was conducted of 37 patients who underwent a total of 130 administrations of ¹⁷⁷Lu-DOTATATE at the Institute of Science Tokyo (formerly Tokyo Medical and Dental University) between April 1, 2022, and April 30, 2025. Clinical information collected included age, sex, activities of daily living (ADL), arrhythmic history, dates and number of administrations, and relevant laboratory and imaging data.

### Clinical and imaging parameters

Vital sign parameters were collected at designated time points: systolic blood pressure, diastolic blood pressure, heart rate, and blood oxygen saturation. The monitoring time points were as follows:

- Upon entering the radioactive isotope administration room.
- At the start of amino acid infusion.
- At the start of ¹⁷⁷Lu-DOTATATE administration.
- At the time of changing the administration rate (10 min after the start of ¹⁷⁷Lu-DOTATATE administration).
- At the end of ¹⁷⁷Lu-DOTATATE administration (30 min later).
- At the time of removing the needle after the end of amino acid infusion.

Tumor-related information was also collected, including primary tumor origin, WHO grade [5, 6], Ki-67 index, and Krenning score [7]. Laboratory results were reviewed for electrolyte levels (sodium, potassium, chloride), hemoglobin, albumin, and total protein levels. The maximum tumor diameter was measured on CT or MRI obtained before treatment. The cardiothoracic ratio was calculated, and the presence of cardiac enlargement was visually assessed. Octreotide scintigraphy using indium (¹¹¹In) pentetreotide (^111^In-Pentetreotide, OctreoScan^®^) was reviewed to evaluate tracer uptake at lesions and in the heart (4 h post-injection), scored using the Krenning method.

### Administration protocol

Initially, all treatments were completed entirely within the radiation-controlled area of our hospital, and a single-route method was used, with the infusion line removed after radionuclide administration before the patient returned to the ward. To allow earlier return, the double-route method was later introduced. In this method, ¹⁷⁷Lu-DOTATATE was administered in the nuclear medicine room within the radiation-controlled area of our hospital, where radioactive waste could be handled safely. After completion of administration, the infusion line was removed with patient consent, and the amino acid infusion was subsequently continued for approximately 3 h in a designated hospital room on the ward with special safety measures.

### Single-route method

An intravenous line was inserted into the left forearm or cubital fossa, followed by intravenous injection of ondansetron. An infusion of amino acid was started at 250 mL/h. ¹⁷⁷Lu-DOTATATE was then infused at 50 mL/h beginning 30 min later. After 10 min, the infusion rate was increased to 200 mL/h, and the total dose was administered over 20 min. The amino acid infusion was continued for approximately 3 h after radionuclide administration.

### Double-route method

An intravenous line was inserted into the left forearm or elbow, and ondansetron was administered intravenously. An infusion of amino acid was started at 250 mL/h. A second line was then placed in the right forearm or cubital fossa concurrently. After 30 min, ¹⁷⁷Lu-DOTATATE was infused at 50 mL. After 10 min, the infusion rate was increased to 200 mL/h. Following completion, the right-sided line was removed, while the amino acid infusion continued for approximately 3 h after returning to the ward.

In all cases, the intravenously administered fluids consisted of 1,000 mL of amino acid solution, approximately 80 mL of saline used for ¹⁷⁷Lu-DOTATATE administration, and approximately 2 mL of ondansetron solution. Patients were also allowed to drink water freely during the procedure; however, retrospective quantification of oral water intake was not feasible.

### Statistical analysis

Repeated-measures analysis of variance (ANOVA) was used to evaluate differences in heart rate and blood pressure at four time points: (i) upon entering the radioactive isotope administration room, (ii) initiation of ¹⁷⁷Lu-DOTATATE administration, (iii) dose-rate adjustment, and (iv) completion of administration. For ANOVA, the significance level for two-tailed tests was set at 0.008, calculated by dividing 0.05 by the number of comparisons (six) according to the Bonferroni correction. Heart rate and blood pressure data were displayed in line graphs using mean values with standard deviations and were analyzed irrespective of treatment cycle, as well as stratified by initial versus subsequent administrations and by tumor grade.

To identify predictors of heart rate change, multiple regression analysis was performed using age, sex, history of arrhythmia, visually assessed cardiomegaly, and serum potassium level as explanatory variables.

Furthermore, to evaluate predictors of bradycardia (heart rate ≤ 50 bpm), baseline characteristics were compared between the bradycardia and non-bradycardia groups during the dose-rate adjustment phase using appropriate statistical tests. Categorical variables (history of arrhythmia, cardiomegaly, and sex) were analyzed using Fisher’s exact test. Serum potassium level, treated as a normally distributed continuous variable, was compared using Student’s t-test, whereas age, which demonstrated a non-normal distribution, was analyzed using the Wilcoxon rank-sum test. The significance level for comparisons between the two groups was set at 0.01, calculated by dividing 0.05 by the number of comparisons (five) according to the Bonferroni correction.

All statistical analyses were performed using MedCalc^®^ (MedCalc Software, Ostend, Belgium) and EZR (Saitama Medical Center, Jichi Medical University, Saitama, Japan), a graphical user interface for R (The R Foundation for Statistical Computing, Vienna, Austria). Statistical analyses were conducted using MedCalc^®^ (Version 23.3.7–64 bit), and figures were generated using EZR on R Commander (Version 1.68, Rcmdr Version 2.9-1.9) [8].

### Patient characteristics

Thirty-seven patients received ¹⁷⁷Lu-DOTATATE therapy, with a total of 130 treatments administered. Of the participants, 20 were male (74 treatments) and 17 were female (56 treatments), with a median age of 59.0 years [interquartile range: 32.0–77.0]. The maximum tumor size measured on CT demonstrated a wide distribution, with a median of 35 mm (interquartile range: 32.0–77.0 mm). Twenty-eight patients (76%) completed the standard treatment course consisting of four cycles administered at 8-week intervals (Table 1).

**Table 1:** Patient and treatment characteristics (130 administrations in 37 patients)

Factor	Group	Overall
Arrhythmia	Absent	120 (92.3%)
	Present	10 (7.7%)
Cardiac enlargement (visual assessment)	Absent	110 (84.6%)
	Present	20 (15.4%)
Neuroendocrine Tumor WHO Grade	Grade 1	28 (21.5%)
	Grade 2	84 (64.6%)
	Grade 3	8 (6.2%)
	Uncertain	10 (7.7%)
Sex	Female	56 (43.1%)
	Male	74 (56.9%)
Age	—	59 [32, 77]
Maximum tumor size (mm)	—	35 [12, 153]
Hemoglobin (g/dL)	—	13.3 (1.5)
Serum albumin (g/dL)	—	4.1 (0.4)
Serum total protein (g/dL)	—	7.0 (0.5)
Serum chloride (mEq/L)	—	105.2 (2.5)
Serum potassium (mEq/L)	—	4.3 (0.4)
Serum sodium (mEq/L)	—	140.9 (2.3)

Categorical variables are presented as n (%). Continuous variables are presented as mean (standard deviation) unless otherwise indicated.

Age and computed tomography–measured maximum tumor size are presented as median [range].

g/dL = grams per deciliter; mEq/L = milliequivalents per liter; mm = millimeters; WHO = World Health Organization.

### Changes in heart rate and blood pressure during administration

**Fig. 1:**
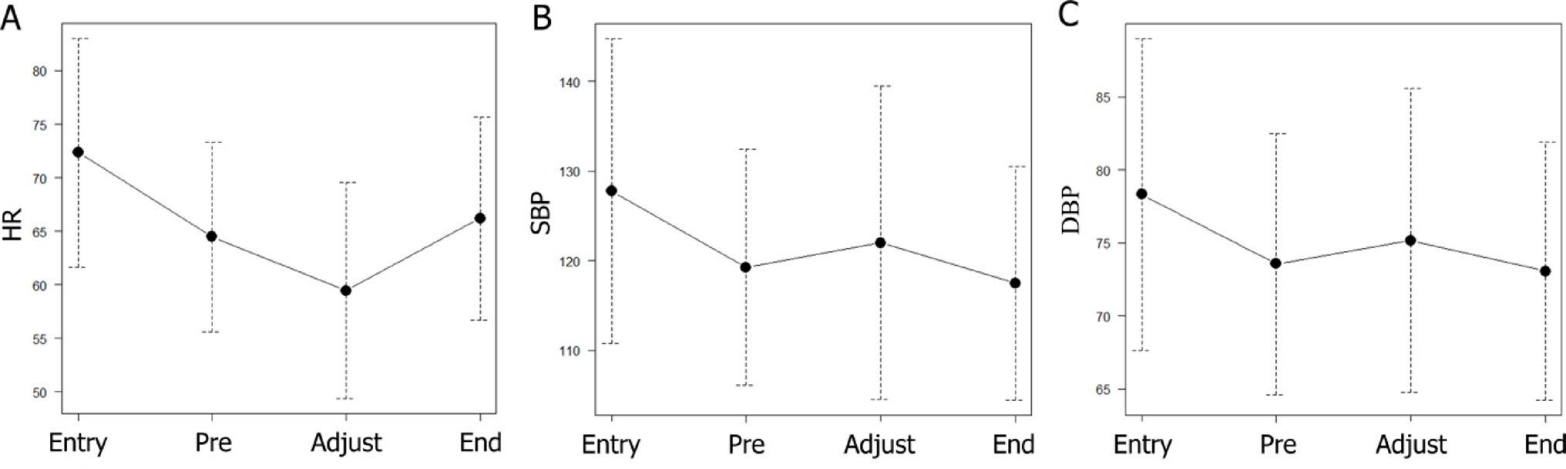
Changes in heart rate and blood pressure over time during medication Dots indicate mean values, and bars represent standard deviations. The mean heart rates at each time point were 72.3, 64.5, 59.5, and 66.2 beats per minute (bpm), respectively. The corresponding mean systolic blood pressure values were 127.8, 119.3, 122.0, and 117.5 mmHg, and the mean diastolic blood pressure values were 78.3, 73.6, 75.2, and 73.1 mmHg, respectively. A gradual decrease in heart rate over time, with a rebound at the end of administration, was observed. Blood pressure also tended to decrease leading up to the administration of ¹⁷⁷Lu-DOTATATE. Abbreviations: HR = heart rate; SBP = systolic blood pressure; DBP = diastolic blood pressure; Entry = upon entering the room; Pre = before ¹⁷⁷Lu-DOTATATE administration; Adjustment = during rate adjustment (10 min after initiation); End = upon completion of administration

Mean heart rate, systolic blood pressure, and diastolic blood pressure were compared at four time points: upon entering the room, before ¹⁷⁷Lu-DOTATATE administration, during dose-rate adjustment, and after completion of ¹⁷⁷Lu-DOTATATE administration. Mean heart rate (72.3 → 64.5 → 59.5 → 66.2 bpm; *p* < 0.0001; Fig. 1A), mean systolic blood pressure (127.8 → 119.3 → 122.0 → 117.5 mmHg; *p* < 0.0001; Fig. 1B), and mean diastolic blood pressure (78.3 → 73.6 → 75.2 → 73.1 mmHg; *p* < 0.0001; Fig. 1C) all showed significant changes.

**Fig. 2:**
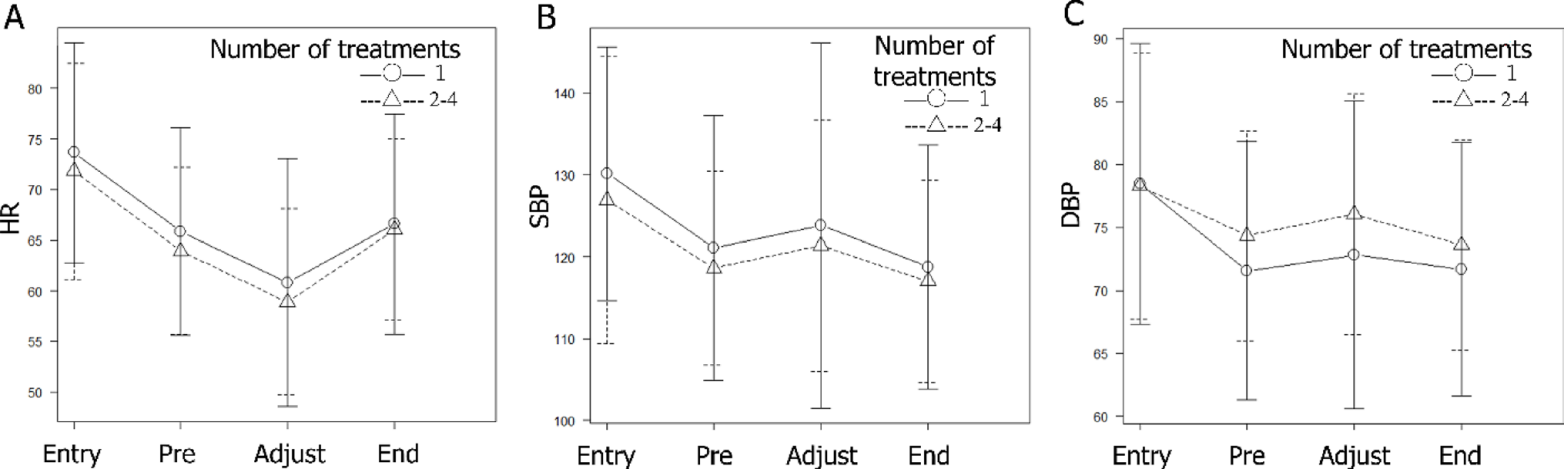
Time-dependent changes in heart rate during ¹⁷⁷Lu-DOTATATE administration: Stratification based on the numbers of treatment **.**Circles indicate the mean values during the first treatment cycle, and triangles indicate the mean values during the 2nd–4th cycles. Solid bars represent standard deviations for the first cycle, and dashed bars indicate standard deviations for the 2nd–4th cycles. No substantial differences were observed in the overall trends of heart rate or blood pressure changes between the first and subsequent (2nd–4th) treatment cycles. Abbreviations: HR = heart rate; SBP = systolic blood pressure; DBP = diastolic blood pressure; Entry = upon entering the room; Pre = before ¹⁷⁷Lu-DOTATATE administration; Adjustment = during rate adjustment (10 min after initiation); End = upon completion of administration

Regarding heart rate changes specifically, mean heart rate was significantly reduced upon entering the room, prior to ¹⁷⁷Lu-DOTATATE administration, and during dose-rate adjustment. Mean heart rate then increased significantly upon completion of ¹⁷⁷Lu-DOTATATE administration, returning to levels similar to those before administration (Fig. 2A). Significant phase-to-phase differences in mean heart rate are summarized in Table 2, including comparisons from entry to pre-administration, pre-administration to dose-rate adjustment, and dose-rate adjustment to the end of infusion.

**Table 2:** Changes in heart rate and blood pressure parameters during each phase of ¹⁷⁷Lu-DOTATATE administration

Category	Phase	Mean	Std. Error	95% CI	Comparison	Mean diff.	P*
HR	Entry	72.3	1.12	70.1 to 74.6	Entry - Pre	7.9	< 0.0001
	Pre	64.5	0.92	62.7 to 66.3	Pre - Adjust	5.0	< 0.0001
	Adjust	59.5	1.05	57.4 to 61.6	Adjust - End	−6.7	< 0.0001
	End	66.2	0.99	64.2 to 68.2	Pre - End	−1.7	0.031
SBP	Entry	127.8	1.71	124.4 to 131.2	Entry - Pre	8.5	< 0.0001
	Pre	119.3	1.32	116.7 to 121.9	Pre - Adjust	−2.7	0.100
	Adjust	122.0	1.76	118.5 to 125.5	Adjust - End	4.5	0.002
	End	117.5	1.31	114.9 to 120.1	Pre - End	1.8	0.169
DBP	Entry	78.3	1.07	76.2 to 80.5	Entry - Pre	4.8	< 0.0001
	Pre	73.6	0.90	71.8 to 75.3	Pre - Adjust	−1.6	0.271
	Adjust	75.2	1.05	73.1 to 77.2	Adjust - End	2.1	0.075
	End	73.1	0.89	71.3 to 74.8	Pre - End	0.5	1

Std. Error: standard error; Mean diff.: mean difference (left phase minus right phase); HR: heart rate (beats per minute, bpm); SBP: systolic blood pressure (mmHg); DBP: diastolic blood pressure (mmHg); CI: confidence interval; Entry: entering the treatment room; Pre: before administration of ¹⁷⁷Lu-DOTATATE; Adjust: during infusion dose rate adjustment; End: at the end of ¹⁷⁷Lu-DOTATATE administration.

*P values correspond to the mean differences and are Bonferroni-corrected for multiple comparisons.

Regarding blood pressure changes, decreases were observed upon entering the room and before ¹⁷⁷Lu-DOTATATE administration (Fig. 2B and C).

The tendency for mean heart rate to decrease before ¹⁷⁷Lu-DOTATATE administration and during dose-rate adjustment, followed by a return to pre-administration levels upon completion of administration, was consistent between the first and the 2nd–4th treatment cycles (Fig. 2A).

Similarly, the tendency for mean blood pressure to decrease prior to ¹⁷⁷Lu-DOTATATE administration did not differ between the first and the 2nd–4th cycles (Fig. 2B and C).

**Fig. 3:**
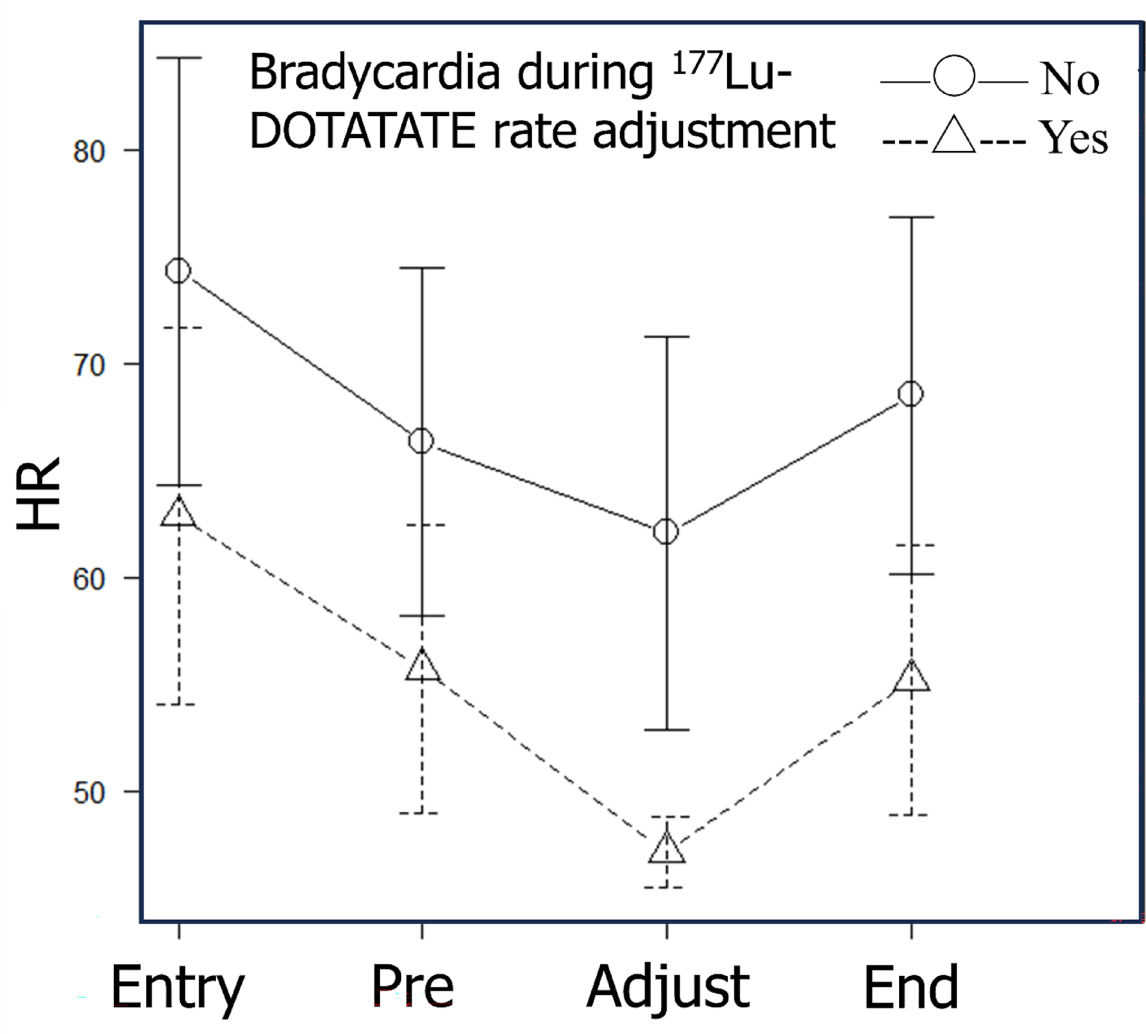
Time-dependent changes in heart rate during ¹⁷⁷Lu-DOTATATE administration: Stratification based on the presence or absence of bradycardia during dose rate adjustment. Mean heart rate values are shown for each phase of administration, with bars representing standard deviations. Patients were divided into two groups based on whether they exhibited bradycardia (heart rate ≤ 50 bpm) during the dose-rate adjustment phase. Patients who developed bradycardia tended to have lower baseline heart rates compared with those who did not exhibit bradycardia. Abbreviations: HR = heart rate; Entry = upon entering the room; Pre = before ¹⁷⁷Lu-DOTATATE administration; Adjustment = during dose-rate adjustment; End = upon completion of administration

In 22 cases, the heart rate fell to ≤ 50 bpm, indicating bradycardia during dose adjustment. Fourteen patients maintained a heart rate ≥ 51 bpm without bradycardia at initiation. Three patients had bradycardia prior to administration, and five had incomplete data. Patients were stratified based on whether they exhibited bradycardia during dose-rate adjustment, and changes in mean heart rate were evaluated. Patients who developed bradycardia tended to have lower baseline heart rates than those who did not (Fig. 3).

**Fig. 4:**
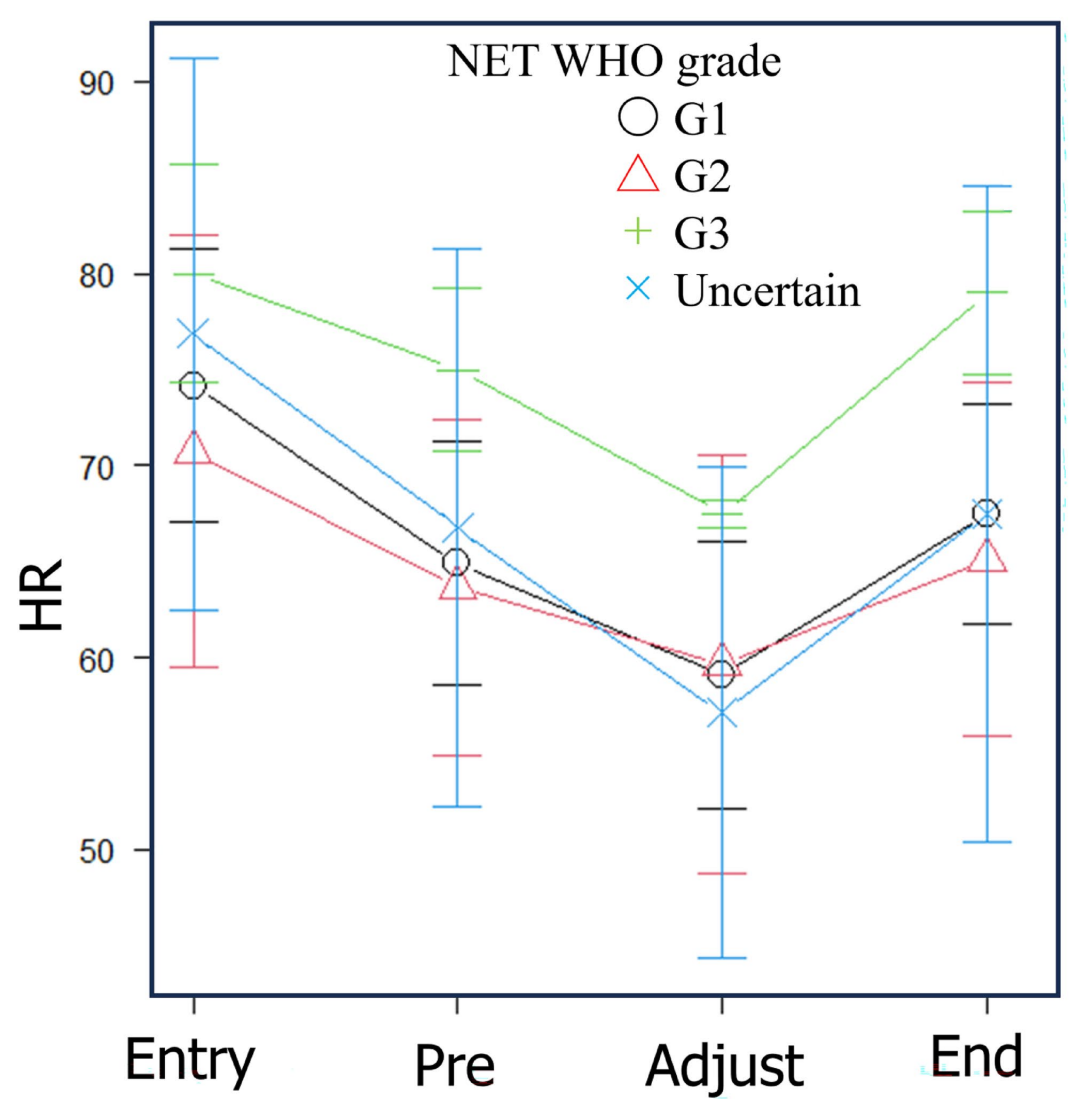
Time-dependent changes in heart rate during medication in patients stratified by World Health Organization (WHO) grade of neuroendocrine tumors. Symbols corresponding to each WHO grade represent mean heart rate values, and the bars indicate standard deviations. Even after stratification by WHO grade, the overall trend in heart rate changes remained unchanged. “Uncertain” refers to cases in which WHO grade information could not be retrospectively obtained

Despite stratification of the cohort by World Health Organization (WHO) grade, a consistent trend was observed: mean heart rate decreased prior to ¹⁷⁷Lu-DOTATATE administration and during dose-rate adjustment, and then returned to pre-administration levels upon completion of administration (Fig. 4).

A total of five patients reported chest discomfort. All five experienced bradycardia both before and after dose-rate adjustment, and in three of these patients, symptoms occurred when the heart rate fell below 50 bpm as recorded by the monitor. However, no decrease in blood pressure was observed at the time of symptom onset.

### Predictors of bradycardia (≤ 50 bpm)

**Fig. 5:**
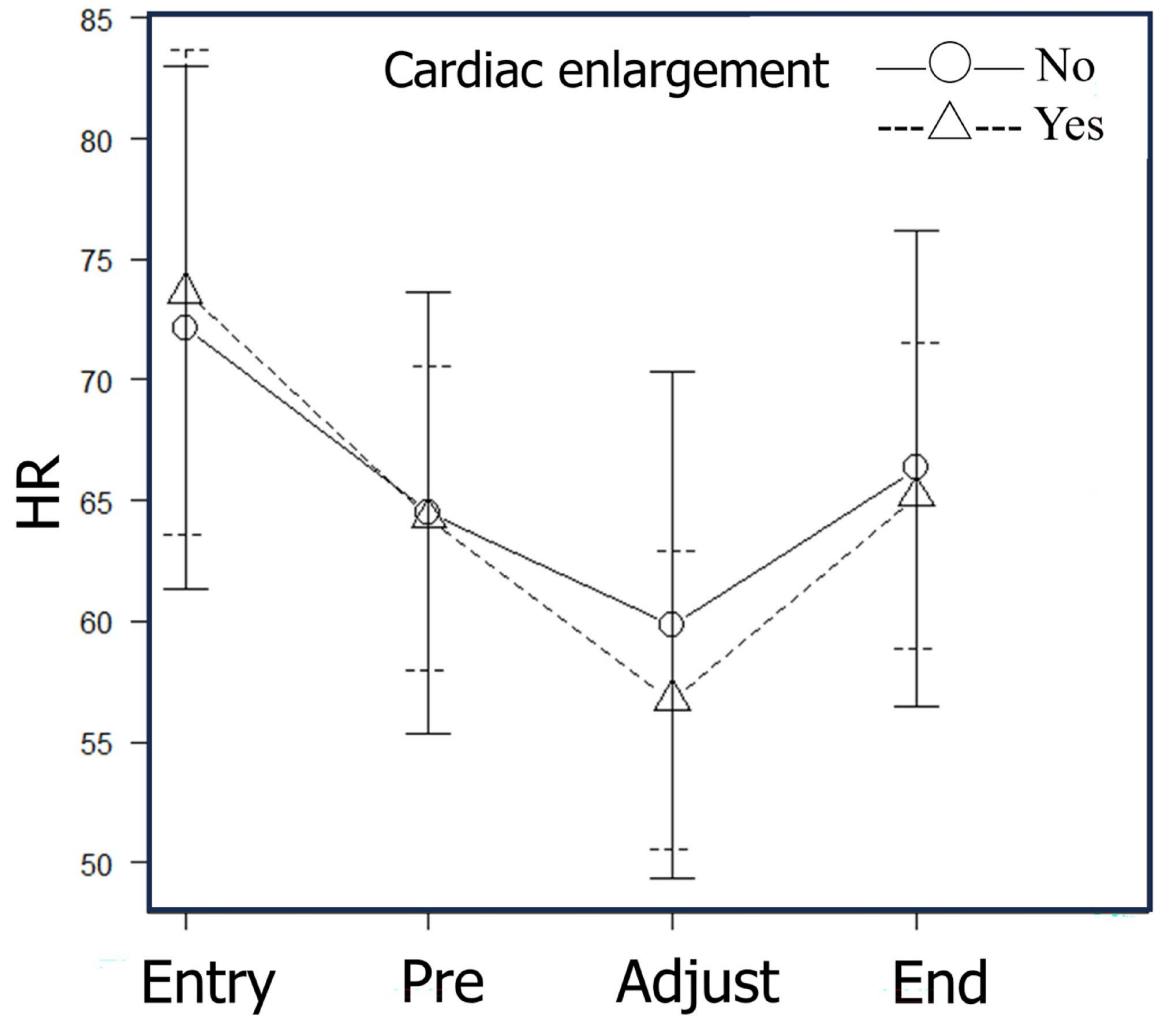
Time-dependent changes in heart rate stratified by the presence or absence of cardiac enlargement determined by visual inspection. Circles represent mean heart rate values in patients with cardiac enlargement, with solid bars indicating standard deviations (SD). Triangles represent mean values in patients without cardiac enlargement, with dashed bars indicating SD. Baseline heart rate did not differ significantly between the two groups; however, patients with cardiac enlargement demonstrated a steeper decline in heart rate during medication. Abbreviations: HR = heart rate; Entry = upon entering the room; Pre = before ¹⁷⁷Lu-DOTATATE administration; Adjustment = during rate adjustment (10 min after initiation); End = upon completion of administration

When comparing cases that developed bradycardia (≤ 50 bpm) during dose-rate adjustment with those that did not, visually assessed cardiac enlargement was the only background factor that showed a low p-value (*p* = 0.045) (Table 3). When stratified by the presence or absence of visually apparent cardiac enlargement, cases with cardiac enlargement demonstrated a greater reduction in mean heart rate during administration (Fig. 5). Although multiple testing precludes definitive conclusions regarding statistical significance, these findings suggest that patients with cardiac enlargement or a higher cardiothoracic ratio may be more susceptible to developing bradycardia during ¹⁷⁷Lu-DOTATATE administration.

**Table 3:** Patient characteristics stratified by the presence or absence of bradycardia during dose rate adjustment (*n* = 121)

Factor	Group	Bradycardia Absent (*n* = 99)	Bradycardia Present (*n* = 22)	*p*-value
Arrhythmia	Absent	92 (92.9%)	20 (90.9%)	0.667
	Present	7 (7.1%)	2 (9.1%)	
Cardiac enlargement	Absent	87 (87.9%)	15 (68.2%)	0.045
	Present	12 (12.1%)	7 (31.8%)	
Sex	Female	44 (44.4%)	9 (40.9%)	0.816
	Male	55 (55.6%)	13 (59.1%)	
Serum potassium (mEq/L), mean (SD)	—	4.30 (0.38)	4.43 (0.36)	0.149
Age, years, median [IQR]	—	59.0 [32.0–77.0]	57.5 [32.0–76.0]	0.721

Values are presented as n (%) for categorical variables, as mean (standard deviation) for normally distributed continuous variables, and as median [interquartile range] for non-normally distributed continuous variables. SD: standard deviation; IQR: interquartile range; mEq/L: milliequivalents per liter. Nine patients were excluded due to missing heart rate data during the dose rate adjustment phase, resulting in 121 patients included in this analysis

## Discussion

The present study demonstrated that during ¹⁷⁷Lu-DOTATATE administration, a transient decrease in heart rate occurred, although values generally returned to baseline by the end of infusion. While a few patients experienced chest symptoms associated with bradycardia, these improved rapidly with observation, suggesting that active intervention is unnecessary unless accompanied by more severe events such as syncope.

In the NETTER-1 trial [1], adverse events of grade 3 or lower were documented, and ECGs were obtained at baseline, immediately after completion of ¹⁷⁷Lu-DOTATATE infusion, and at the end of the study. In contrast to our protocol, ECGs were not performed during infusion. Therefore, transient bradycardia occurring during administration would not have been detected, consistent with the absence of reported events in that trial.

High-dose L-arginine administration is known to lower blood pressure and heart rate [9–11]. The high-concentration amino acid infusion used with ¹⁷⁷Lu-DOTATATE contains 25 g of L-arginine and 25 g of L-lysine . According to the report by Saqib et al. [3], administration of 30 g of L-arginine at a rate of 1 g/min resulted in decreased blood pressure and heart rate (prolonged RR interval), in contrast to the blood pressure decrease observed in the control group due to hydralazine. The proposed mechanism involves L-arginine–induced autonomic modulation through nitric oxide (NO) production [1]. In our protocol, L-arginine was infused at a rate of 25 g over 240 min, equivalent to approximately 0.1 g/min. Although this concentration is lower than that used by Saqib et al., it is plausible that similar NO-mediated autonomic effects contributed to the observed decrease in heart rate.

Among patients with cardiac enlargement, baseline heart rate did not differ from that of patients without enlargement; however, a greater reduction in heart rate was observed during dose-rate adjustment. In patients with heart failure, L-arginine substrate deficiency and altered responsiveness to arginine and NO have been described by Mendes Ribeiro et al. [12]. It is therefore possible that similar alterations in L-arginine responsiveness during high-concentration amino acid infusion may exist even in patients with isolated cardiac enlargement.

The potential influence of ondansetron was also considered. Ondansetron has been used as prophylaxis against hypotension during spinal epidural anesthesia. Although a randomized controlled trial by Owczuk et al. [13] demonstrated its hypotension-preventive effect, no change in heart rate was reported. Given its pharmacologic profile, the decreases in heart rate and blood pressure observed in our study are unlikely to be attributable to ondansetron.

We also considered whether fluid administration might have contributed to the decrease in heart rate. However, no correlation was observed between changes in heart rate and blood pressure. Moreover, at the highest infusion rate of 200 mL/h, heart rate actually increased. These findings collectively suggest that a simple volume-loading effect is unlikely to explain the observed heart rate decrease.

Vagal nerve reflexes triggered by puncture or changes in posture are known to modulate heart rate [14]. In our cohort, heart rate was measured at the start of ¹⁷⁷Lu-DOTATATE infusion, at which point the amino acid infusion (L-lysine hydrochloride and L-arginine hydrochloride) had already been running for more than 15 min, well after venipuncture. Therefore, in the comparison between the initiation of ¹⁷⁷Lu-DOTATATE administration and the dose-rate adjustment phase, the influence of any vagal reflex associated with venipuncture was considered negligible. Patients were kept in a supine position throughout administration; only one patient stood briefly during urination, suggesting that the impact of body position or activity on heart rate was minimal.

Chest symptoms due to vagal reflexes or allergic reactions to ¹⁷⁷Lu-DOTATATE or the amino acid infusion were also considered. Chest symptoms occurred in five cases; however, no significant changes in blood pressure were observed at the time of symptom onset. Therefore, a vagal reflex was considered an unlikely cause of these symptoms. Four patients developed localized skin rashes at the puncture site, but their heart rates during infusion adjustments (49, 63, 69, and 82 bpm) were not remarkably abnormal, indicating limited clinical influence.

The potential role of radiation exposure was also explored. Alter et al. demonstrated in rats that exposure to high-energy electron beams (≥ 10 Gy) induced transient decreases in blood pressure and heart rate, attributed to autonomic dysfunction [15]. However, the estimated myocardial wall absorbed dose of ¹⁷⁷Lu-DOTATATE is only 0.032 Gy/GBq according to FDA prescribing information [4], well below the threshold observed in animal models. While late effects of low-dose radiation exposure, such as fibrosis, have been associated with the development of bradycardia during long-term follow-up, the occurrence of acute, transient bradycardia immediately after radionuclide infusion has neither been reported nor is it mechanistically plausible [16–18]. Therefore, radiation itself is unlikely to account for the transient bradycardia observed in this study. The underlying mechanism thus remains uncertain.

By comparing the bradycardia and non-bradycardia groups, we identified cardiac enlargement as a risk factor for bradycardia during ¹⁷⁷Lu-DOTATATE infusion. Although the physiological basis of this association remains uncertain, awareness of this relationship is important for appropriate clinical monitoring.

In addition, a recent post-marketing pharmacovigilance study analyzing 4,284 Lutathera-related adverse event reports from the FDA Adverse Event Reporting System (FAERS) revealed high overall mortality and significant signals across multiple system organ classes, particularly endocrine disorders [19]. Importantly, however, cardiovascular events such as bradycardia were not highlighted in this large-scale dataset. Taken together with the findings from the NETTER-1 trial [1] and the FDA label [4], which also do not describe heart rate alterations, our results suggest that transient bradycardia is a benign phenomenon detectable mainly under continuous monitoring conditions, rather than a widely recognized adverse event in routine clinical practice.

## Limitations

This was a single-center retrospective study, and the potential influence of institutional practice or environmental factors cannot be excluded. The lack of a strict temporal framework for vital sign measurements limits the precision of the temporal relationship between infusion events and observed bradycardia. Vital signs immediately prior to amino acid infusion could be retrospectively obtained in only 10 of 130 cases, indicating a high proportion of missing data. Consequently, when evaluating the effects of the amino acid infusion, it was not possible to compare vital signs immediately before amino acid infusion with those immediately before ¹⁷⁷Lu-DOTATATE administration. Vital signs recorded upon entering the room were therefore used as a surrogate; however, in this context, it was difficult to exclude the potential influence of puncture-related stress or activities such as walking. In addition, differences in infusion volume between single- and double-route administration likely affected the timing of systemic drug exposure, but this could not be evaluated retrospectively.

Heart rate can also vary considerably with physical activity and sleep. Healthy individuals may experience transient decreases in heart rate to 30–35 bpm during sleep [20]. However, such information could not be systematically collected in this retrospective study.

Furthermore, this study did not include dosimetric assessment, and therefore the influence of absorbed radiation dose to the myocardium could not be precisely evaluated. In addition, tracer uptake was assessed using OctreoScan^®^, which is less sensitive than post-therapy scans [21] or positron emission tomography (PET) tracers such as gallium (⁶⁸Ga) oxodotreotide (⁶⁸Ga-DOTATATE) [22]. Consequently, tumor and cardiac uptake may have been underestimated, leaving room for further evaluation of the association between cardiac uptake and bradycardia using more sensitive imaging modalities.

## Conclusions

This study is the first to retrospectively characterize transient bradycardia occurring during radionuclide therapy with ¹⁷⁷Lu-DOTATATE, to identify potential risk factors, and to evaluate the need for intervention. We found that bradycardia was more likely in patients with cardiac enlargement, whereas no other significant predictors were identified. Importantly, these episodes were transient, asymptomatic, and resolved spontaneously, indicating that unnecessary treatment interruption is generally unwarranted. Awareness of this benign nature, together with recognition of cardiac enlargement as a risk factor, may help reduce confusion in the clinical setting and ensure safe and uninterrupted therapy.

## Supplementary Information

Below is the link to the electronic supplementary material.

Supplementary Material 1

## Data Availability

The datasets generated and/or analyzed during the current study are available from the corresponding author upon reasonable request.

## Abbreviations

HR: heart rate
Entry: upon entering the room
Pre: before ¹⁷⁷Lu-DOTATATE administration
Adjustment: during rate adjustment (10 min after initiation)
End: upon completion of administration
