# Supplementary figures and images for: Transient bradycardia during 177Lu-DOTATATE therapy: A clinically manageable phenomenon with increased risk in patients with cardiac enlargement
Source: Ann Nucl Med. 2025 Dec 29;40(5):496–506. doi: 10.1007/s12149-025-02150-4 (PMC13124752; doi:10.1007/s12149-025-02150-4)

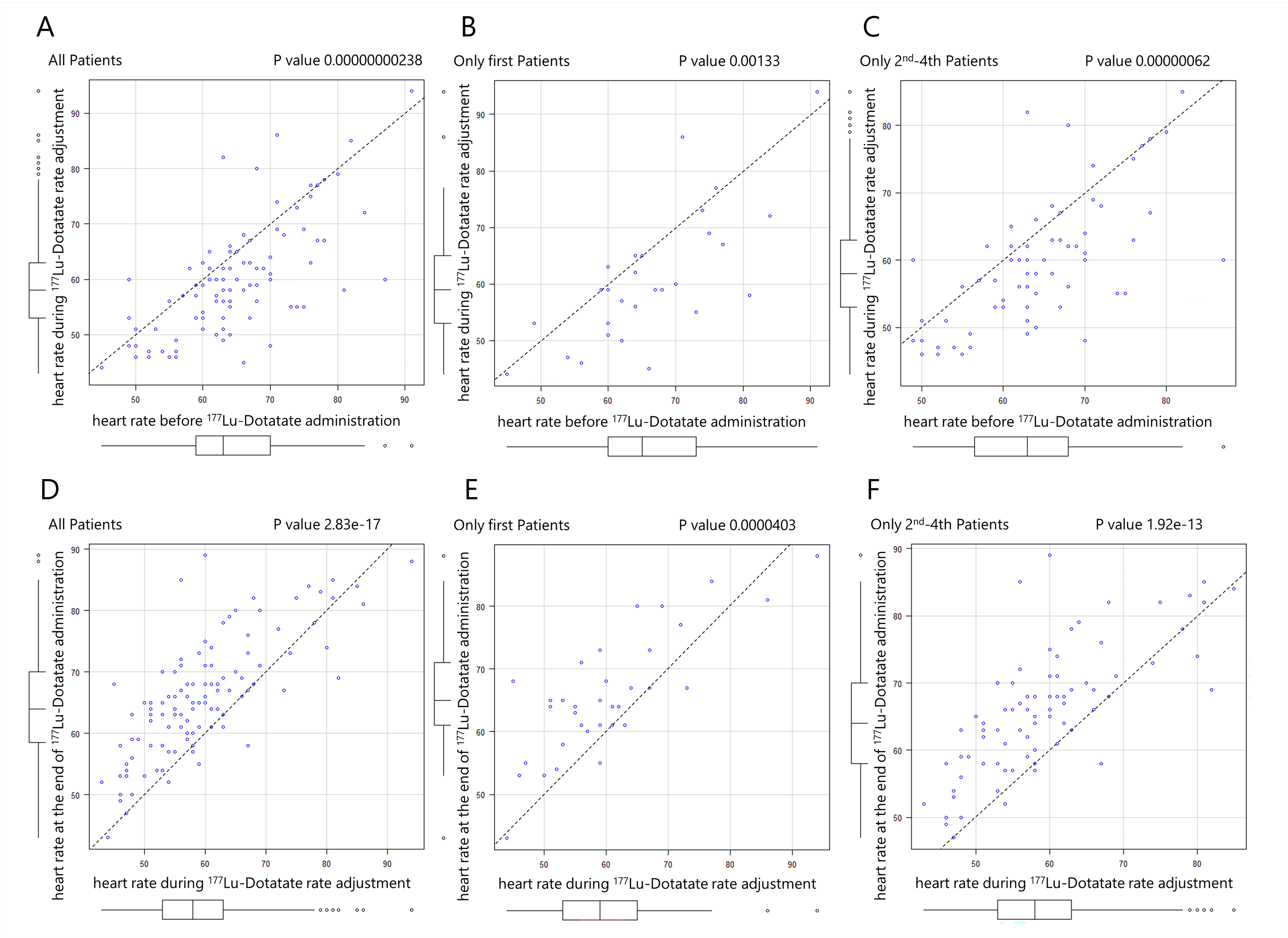

Supplement: Supplementary file 1 — Supplementary Material 1 [file 12149_2025_2150_MOESM1_ESM.tif]
